# Distributed Consensus Control for Discrete-Time T–S Fuzzy Multiple-Agent Systems Based on an Unknown Input Observer

**DOI:** 10.3390/s24248149

**Published:** 2024-12-20

**Authors:** Xufeng Ling, Haichuan Xu, Weijie Weng, Fanglai Zhu

**Affiliations:** 1 School of Artificial Intelligence, Shanghai Normal University Tianhua College, Shanghai 201815, China; lxf1131@sthu.edu.cn; 2 Shanghai Research Institute for Intelligent Autonomous Systems, Tongji University, Shanghai 200092, China; xu_hai_chuan@163.com; 3 School of Electronics and Information Engineering, Tongji University, Shanghai 201804, China; 2130742@tongji.edu.cn

**Keywords:** T–S fuzzy systems, multiple-agent systems, unknown input observer, distributed consensus, zonotope theory

## Abstract

This paper investigates a consensus problem for a class of T–S fuzzy multiple-agent systems (MASs) with unknown input (UI). To begin with, an unknown input observer (UIO) is able to asymptotically estimate the system state and the UI is designed for each agent. In order to construct the UIO, the state interval estimation is obtained by first using zonotope theory. Next, using the interval estimation of the state, a correlation of the state and the UI is built. Subsequently, a UIO is constructed, which is proposed by building upon the algebraic relationship. Moreover, by using the estimations of the state and the UI, a distributed control protocol is developed based on the proposed UIO. And, with the proposed distributed control protocol, the T–S fuzzy MAS can achieve consensus, in that all the states of the agents can converge to the leader’s state asymptotically. Finally, the effectiveness of the proposed method is demonstrated through two simulation examples.

## 1. Introduction

Recently, cooperative control has drawn much attention, since its extensive applications include all kinds of fields such as formation [1,2,3], flocking [4,5], containment [6,7], and consensus [8,9]. The dominant principle of the distributed consensus control of MASs aims to achieve agreement on a common state value among agents by designing a distributed control protocol through information interaction, where one of the major control objectives is to guarantee the synchronization of the agents’ state trajectories. The consensus control of MASs can be categorized in two regiments. They are leaderless structure and the leader–follower configuration. So far, many research results related to handling MAS consensus control problems have been given in the literature [10,11,12,13,14]. In [10], sufficient conditions were presented for guaranteeing the leaderless consensus of linear MASs with fixed information communication topology. The leaderless consensus control problems of linear MASs having feedback delay were discussed in [11,12], while, in [13], the consensus control problems for discrete-time MASs, which have communication delays, were dealt with. The fixed-time leader-following consensus issues were investigated for MASs with discontinuous dynamics in [14]. It should be emphasized that most discussions on MAS cooperative control, especially for consensus control problems, are in relation to linear systems, and little work has been carried out for nonlinear MASs [15,16,17,18]. The fixed-time consensus of heterogeneous nonlinear MASs was investigated in [15]. The event-triggered adaptive tracking problem was solved for nonlinear switching MASs in [16]. In [17], the finite-time lag consensus was studied for nonlinear MASs by applying finite-time passivity. The adaptive output consensus of heterogeneous nonlinear MASs was investigated by using a distributed dynamic compensator approach in [18].

The concept of a T–S fuzzy model was first proposed in [19]. In the model, a nonlinear system is characterized by a series of linear subsystems that are obtained by locally linearizing the original nonlinear system at some specific operating points. As a result, the model is actually a special kind of nonlinear system in which the nonlinearity is focally reflected in the nonlinear weight functions. In fact, nonlinear systems can be approximated by T–S fuzzy systems in any convex compact region to any accuracy degree [20]. Therefore, coping with nonlinear systems becomes convenient by using T–S fuzzy models, and, consequently, they have attracted many researchers’ attention. For example, in [21], asymptotic convergent tracking problems were investigated for uncertain switched nonlinear systems with a fuzzy approximation framework. In [22], a discrete-time T–S fuzzy system with uncertainty was considered for dealing with fault detection and isolation problems. In [23], a T–S fuzzy fractional-order nonlinear nonautonomous system was considered with input delay. In [24], fault-tolerant control was investigated for fuzzy systems with different faults. Although T–S fuzzy models have played an important role in handling control problems for a single nonlinear system, the MAS control problems for T–S fuzzy systems are still open and deserve to be investigated deeply. In fact, up to the present, very few discussions on this issue can be found in the literature [25,26]. In [25], the asynchronous tracking problem in T–S fuzzy MASs with general dynamics was examined through the application of an event-triggering mechanism. Upon considering T–S fuzzy models within the context of nonlinear MASs, the issue of achieving leader-following output consensus in nonlinear switched T–S fuzzy MASs was examined in [26].

In practical applications, many factors have to be considered in cooperative control designs of single-agent system and MASs, including unpredictable external disturbances, actuator and sensor faults, noise impacting the measurement output, and so on, which can all be due to unknown inputs to the systems. Consequently, different observers that contain the Luenberger observer and UIOs, which are constructed to estimate the system states or even to obtain an unknown input reconstruction (UIR), are introduced into cooperative control designs. In fact, the problems of observer-based consensus control have gained intensive attention from scholars [27,28,29,30,31,32,33,34,35,36,37,38]. For instance, a zonotopic observer was designed in [27] for uncertain T–S fuzzy single-agent systems. A distributed observer-based H-infinity control protocol was presented in [28] for general MASs subject to noise and unknown inputs. The problem of the observer-based event-triggered tracking control for nonlinear MASs under DoS attacks was addressed in [33]. Under a DoS attack environment and for nonlinear MASs, the issue of observer-based event-triggered tracking control was examined in [33]. In [34], dynamic event-triggered bipartite consensus in MASs under a competitive network was investigated and an observer-based control protocol was proposed. In [36], an asymptotic consensus control scheme was worked out for MASs with unknown disturbances by designing a UIO. In [37], the consensus control problem was addressed for MASs with unknown inputs (UIs) and external disturbances.

In the present paper, the UIO-based consensus control problems for a class of T–S fuzzy MASs with unknown inputs are investigated. The major contributions of the paper are as follows:

(1) For a T–S fuzzy system with UI, an algebraic correlation of the state and the UI is established. To reach this goal, an iterative calculating method for the state and then the output interval estimations are developed via zonotope theory;

(2) Through the combination of a Luenberger-like observer together with a UIR, a UIO design method is put forward, wherein the UIR is given by using the proposed algebraic relationship. Consequently, the proposed UIO is able to obtain the estimations of both the states and the UI asymptotically;

(3) Utilizing the state estimation and the UIR, a distributed control protocol is schemed by using the proposed UIO, and, under this distributed protocol, the T–S fuzzy MAS in leader–follower constructure achieves consensus asymptotically, even if it suffers from UIs.

The paper is arranged in this way. Preliminaries are presented in Section 2. In Section 3, the interval estimation of the state bases on zonotope theory and then the UIO design are carried out. The distributed control protocol is developed in Section 4. Section 5 provides a simulation example to demonstrate the performances of the proposed methods. In Section 6, some conclusions are summarized.

## 2. Preliminaries

In this section, some notations are listed. Additionally, the descriptions of the MASs along with the preconditions are presented.

### 2.1. Notations

$R^{n}$ and $R^{n\times m}$ denote the set of *n*- and n×m-dimensional vectors and matrices, respectively. diag(·) is a diagonal matrix. ∥·∥ is the Euclidean norm. For any $M=\left[m_{ij}\right]\in R^{n\times m}$, define $M^{+}=\left[m_{ij}^{+}\right]$, $M^{-}=\left[m_{ij}^{-}\right]$, and $\left|M\right|=\left[\left|m_{ij}\right|\right]$, with $m_{ij}^{+}=\max\left(0,m_{ij}\right)$ and $m_{ij}^{-}=\max\left(0,-m_{ij}\right)$. Thus, $M=M^{+}-M^{-}$ and $\left|M\right|=M^{+}+M^{-}$.

### 2.2. Graph Theory

The communication topology is expressed by a directed graph G=(V,W,A), with V={1,⋯,N},W⊆V×V, and $A=\left[a_{ij}\right]$ acting as, respectively, the sets of nodes and edges, and the adjacency matrix. (i,j)∈W means extracting a message from j→i and, thus, node *j* becomes a neighbor of node *i*, and, correspondingly, we set $a_{ij}>0$; otherwise, we set $a_{ij}=0$. In addition, we have $a_{ii}=0$. We define a Laplacian matrix as $L=\left[l_{ij}\right]$, with $l_{ii}=\sum_{j=1}^{N}a_{ij}\left(i\in V\right)$ and $l_{ij}=-a_{ij}$ for i≠j. Denote $B=\mathrm{diag}\left(b_{1},\cdots ,b_{N}\right)$; $b_{i}>0$⇔ the agent *i* is a neighbor of the leader, and $b_{i}=0$ otherwise. After introducing $C=\mathrm{diag}\left(\sum_{j=1}^{N}a_{1j},\cdots ,\sum_{j=1}^{N}a_{Nj}\right)$, we have L=C−A. Moreover, define H=L+B. If we denote $H=\left[h_{ij}\right]\in R^{N\times N}$, then we have $\sum_{j=1}^{N}h_{ij}=b_{i}$.

### 2.3. MAS Model

The agent i(i∈V) is a T–S fuzzy system formulated by a class of IF–THEN fuzzy rules.

**Plant Rule** $f_{i}$: IF $w_{i1}\left(\tau \right)$ is $F_{f_{i}1}$, $w_{i2}\left(\tau \right)$ is $F_{f_{i}2}\ldots$, and $w_{i\Lambda }\left(\tau \right)$ is $F_{f_{i}\Lambda }$, $f_{i}=1,\cdots ,l$,

THEN

$$\begin{array}{c} \left\{\begin{array}{c} x_{i}\left(\tau +1\right)=A_{f_{i}}x_{i}\left(\tau \right)+B_{f_{i}}\left(u_{i}\left(\tau \right)+v_{i}\left(\tau \right)\right)\\ y_{i}\left(\tau \right)=Cx_{i}\left(\tau \right) \end{array}\right. \end{array}$$

Here, $x_{i}\left(\tau \right)\in R^{n},u_{i}\left(\tau \right)\in R^{l},y_{i}\left(\tau \right)\in R^{p}$, and $v_{i}\left(\tau \right)\in R^{q}$ are the state, control input, output, and unknown input of agent *i*. $w_{i}\left(\tau \right)={\left[\begin{array}{ccc} w_{i1}^{T}\left(\tau \right) & \cdots & w_{i\Lambda }^{T}\left(\tau \right) \end{array}\right]}^{T}$ is the premise vector. $F_{f_{i}j}$ is a fuzzy set defined by the membership function $h_{f_{i}j}\left(w_{i}\left(\tau \right)\right)\left(j=1,\cdots ,\Lambda \right)$, where $A_{f_{i}},B_{f_{i}}$, and *C* are constant matrices. Through the center-average defuzzifier, one has

(1) $$\begin{array}{c} \left\{\begin{array}{c} x_{i}\left(\tau +1\right)=A\left(\varpi \left(\tau \right)\right)x_{i}\left(\tau \right)+B\left(\varpi \left(\tau \right)\right)\left(u_{i}\left(\tau \right)+v_{i}\left(\tau \right)\right)\\ y_{i}\left(\tau \right)=Cx_{i}\left(\tau \right) \end{array}\right. \end{array}$$

where $A\left(\varpi \left(\tau \right)\right)=\sum_{f_{i}=1}^{l}\varpi_{f_{i}}\left(w_{i}\left(\tau \right)\right)A_{f_{i}},B\left(\varpi \left(\tau \right)\right)=\sum_{f_{i}=1}^{l}$$\varpi_{f_{i}}\left(w_{i}\left(\tau \right)\right)B_{f_{i}}$, with $\varpi_{f_{i}}\left(w_{i}\left(\tau \right)\right)=\prod_{j=1}^{\Lambda }h_{f,j}\left(w_{i}\left(\tau \right)\right)/\sum_{f_{i}=1}^{l}\prod_{j=1}^{\Lambda }h_{f_{ij}j}\left(w_{i}\left(\tau \right)\right)$ being the fuzzy weights. And the leader agent is formulated by

(2) $$\begin{array}{c} x_{0}\left(\tau +1\right)=A\left(\varpi \left(\tau \right)\right)x_{0}\left(\tau \right) \end{array}$$

**Remark** **1.** *In [29], observer-based $H_{\infty }$ drive–response synchronization was investigated for switched nonlinear time-delay systems with random injection attacks, while, in the present paper, distributed consensus issue is discussed for T–S fuzzy MASs based on a UIO. Both papers focus on system state estimation with different observers. Differently from [29], where the response system is subject to disturbance belonging to $L_{2}\left[0,+\infty \right)$, the disturbance in the present paper is assumed to be bounded and its boundary is known; under this assumption, a UIO is designed to estimate it asymptotically. After this, a distributed controller for each agent is designed by using the unknown input reconstruction to reach asymptotic convergence MAS consensus.*

**Assumption** **1.** *We assume that ${\underline{x}}_{i,0}\leq x_{i}\left(0\right)\leq {\bar{x}}_{i,0}$ and ${\underline{v}}_{i}\leq v_{i}\left(\tau \right)\leq {\bar{v}}_{i}$; here, ${\underline{x}}_{i,0},{\bar{x}}_{i,0},{\underline{v}}_{i}$, and ${\bar{v}}_{i}$ are all known constant vectors.*

**Assumption** **2.** *The pairs $\left(A_{f_{i}},B_{f_{i}}\right)$ and $\left(A_{f_{i}},C\right)$ are controllable and observable, respectively. Additionally, the observer matching condition $\mathrm{rank}\left(CB_{f_{i}}\right)=\mathrm{rank}\left(B_{f_{i}}\right)$ holds.*

**Assumption** **3.** *The undirected graph is connected.*

**Definition** **1.** *Denote $B^{s}={\left[-1\;1\right]}^{s}\left(s>=n\right)$, which is a unit hypercube; then, zonotope Z with n-dimension and s-order is an affine transformation of $B^{s}$, defined by $Z=c⊕\Theta B^{s}=\left\{c+\Theta z|z\in B^{s}\right\}$, where vector $c\in R^{n}$ is the center and $\Theta \in R^{n\times s}$ is the generator matrix. To simplify the notation, we employ the symbol Z=〈c,Θ〉 to represent the zonotope.*

For the concept of zonotope, it has the following properties.

**Property** **1.**
$$\begin{array}{c} \begin{array}{cc} \langle c_{1},\Theta_{1}\rangle ⊕\langle c_{2},\Theta_{2}\rangle & =\langle c_{1}+c_{2},\left[\Theta_{1},\Theta_{2}\right]\rangle \\ L⊙\langle c,\Theta \rangle & =\langle Lc,L\Theta \rangle \end{array} \end{array}$$

*where $c,c_{1},c_{2}\in R^{n}$, and $\Theta ,\Theta_{1},\Theta_{2}$, and L are constant matrices.*

**Property** **2.** *For $Z=c⊕\Theta B^{s}\subset R^{n}$, $\bar{\Theta }$ is obtained by rearranging the columns of Θ in descending order of their norms. And $Z\subseteq \langle c,R_{d}\left(\Theta \right)\rangle \left(n\leq d\leq s\right)$, where $R_{d}\left(\Theta \right)=\left[\begin{array}{cc} \Theta^{a} & \Theta^{b} \end{array}\right]\in R^{n\times d};\Theta^{a}$ is comprised of the initial d−n columns of $\bar{\Theta }$, while $\Theta^{b}\in R^{n}$ is a diagonal matrix with $\Theta_{i}^{b}=\sum_{j=d-n+1}^{s}\left|{\bar{\Theta }}_{i,j}\right|,\left(i=1,\cdots ,n\right)$ serving as its diagonal elements. Here, ${\bar{\Theta }}_{i,j}$ stands for the ij th entry of matrix $\bar{\Theta }$.*

**Property** **3.** *For $Z=c⊕\Theta B^{s},\forall x={\left[\begin{array}{ccc} x_{1} & \ldots & x_{n} \end{array}\right]}^{T}$∈Z, we have $c_{i}-\sum_{j=1}^{s}\left|\Theta_{ij}\right|\leq x_{i}\leq c_{i}+\sum_{j=1}^{s}\left|\Theta_{ij}\right|,\left(i=1,\ldots ,n\right)$, which can be written compactly as*

(3) $$\begin{array}{c} c-\hat{\Theta }\leq x\leq c+\hat{\Theta } \end{array}$$

*where $c={\left[\begin{array}{ccc} c_{1} & \ldots & c_{n} \end{array}\right]}^{T}\in R^{n}$ becomes the center of Z, $\hat{\Theta }={\left[\sum_{j=1}^{s}|\Theta_{1j}|\;\cdots \;\sum_{j=1}^{s}\left|\Theta_{nj}\right|\right]}^{T}$, with $\Theta_{ij}$ being the ij th entry of the matrix Θ.*

**Property** **4.** *For any $d\in R^{n}$ satisfying $\underline{d}\leq d\leq \bar{d}$, where $\underline{d},\bar{d}\in R^{n}$, then $d\in Z_{d}=$$c_{d}⊕\Theta_{d}B^{n}=\langle c_{d},\Theta_{d}\rangle$, where $c_{d}=\frac{1}{2}\left(\underline{d}+\bar{d}\right)$ and $\Theta_{d}=\frac{1}{2}\mathrm{diag}\left(\bar{d}-\underline{d}\right)$.*

**Lemma** **1** ([22])**.** *If $\underline{v}\left(\tau \right),v\left(\tau \right)$, and $\bar{v}\left(\tau \right)$ satisfy $\underline{v}\left(\tau \right)\leq v\left(\tau \right)\leq \bar{v}\left(\tau \right)$, then, for any matrix T, we have $T^{+}\underline{v}\left(\tau \right)-T^{-}\bar{v}\left(\tau \right)\leq Tv\left(\tau \right)$$\leq T^{+}\bar{v}\left(\tau \right)-T^{-}\underline{v}\left(\tau \right)$.*

**Definition** **2.** *The consensus of MASs (1) and (2) is fulfilled if $\lim_{\tau \to \infty }∥x_{i}\left(\tau \right)-x_{0}\left(\tau \right)∥=0,\left(i\in V\right)$.*

## 3. UIO Design Based on State Interval Estimation

In this section, firstly, by the combination of a Luenberger-like observer and zonotope theory, a state interval estimation calculation method is offered. Secondly, by using the state interval estimation, an algebraic correlation of the UI and the state is built. Thirdly, by referring to the algebraic relationship, a UIO with a UIR is constructed, and the UIO can offer the estimates of both the state and the UI.

Consider

(4) $$\begin{array}{cc} {\hat{x}}_{i}\left(\tau +1\right) & =A\left(\varpi \left(\tau \right)\right){\hat{x}}_{i}\left(\tau \right)+B\left(\varpi \left(\tau \right)\right)u_{i}\left(\tau \right)+L\left(\varpi \left(\tau \right)\right)\left(y\left(\tau \right)-C{\hat{x}}_{i}\left(\tau \right)\right) \end{array}$$

here, ${\hat{x}}_{i}\left(\tau \right)\in R^{n}$ stands for the state estimation and $L\left(\varpi \left(\tau \right)\right)=\sum_{f_{i}=1}^{l}\varpi_{f_{i}}\left(w_{i}\left(\tau \right)\right)L_{f_{i}}\in R^{n\times p}$, where $L_{f_{i}}\left(f_{i}=1,\cdots ,m\right)$ are constant gain matrices to be determined later. Subtracting (4) from (1) yields

(5) $$\begin{array}{c} {\tilde{x}}_{i}\left(\tau +1\right)=A_{L}\left(\varpi \left(\tau \right)\right){\tilde{x}}_{i}\left(\tau \right)+B\left(\varpi \left(\tau \right)\right)v_{i}\left(\tau \right) \end{array}$$

Here, we denote ${\tilde{x}}_{i}\left(\tau \right)=x_{i}\left(\tau \right)-{\hat{x}}_{i}\left(\tau \right)$ and $A_{L}\left(\varpi \left(\tau \right)\right)=A\left(\varpi \left(\tau \right)\right)-L\left(\varpi \left(\tau \right)\right)C=\sum_{f_{i}=1}^{l}\varpi_{f_{i}}\left(w_{i}\left(\tau \right)\right)\left(A_{f_{i}}-L_{f_{i}}C\right)$. The time variable τ is omitted if no confusion is raised.

**Lemma** **2.** *If the LMIs*

(6) $$\begin{array}{c} \left[\begin{array}{cc} -\Sigma_{i} & *\\ A_{f_{i}}-\Lambda_{i}C & -\Sigma_{i} \end{array}\right]<0,\left(f_{i}=1,\cdots ,l\right) \end{array}$$

*have a solution for symmetric positive definite matrix $\Sigma_{i}\in R^{n}$ and matrix $\Lambda_{i}\in R^{n\times p}$, then (i) when $v_{i}\left(\tau \right)=0$, the observer error system in (5) is asymptotically stable; (ii) when $v_{i}\left(\tau \right)\neq 0,{\tilde{x}}_{i}\left(\tau \right)$ is bounded ⇔$v_{i}\left(\tau \right)$ is bounded.*

**Proof.**  Choose Lyapunov function $V_{a}\left(\tau \right)={\tilde{x}}_{i}^{T}\left(\tau \right)\Sigma_{i}{\tilde{x}}_{i}\left(\tau \right)$; then, when $v_{i}\left(\tau \right)=0$, the difference of it along the trajectory of (5) is

$$\begin{array}{c} \Delta V_{a}\left(\tau \right)={\tilde{x}}_{i}^{T}\left(\tau \right)\left[A_{L}^{T}\left(\varpi \left(\tau \right)\right)\Sigma_{i}A_{L}\left(\varpi \left(\tau \right)\right)-\Sigma_{i}\right]{\tilde{x}}_{i}\left(\tau \right) \end{array}$$

By Schur complement lemma, $A_{L}^{T}\left(\varpi \left(\tau \right)\right)\Sigma_{i}A_{L}\left(\varpi \left(\tau \right)\right)-\Sigma_{i}<0$, which implies that $\Delta V_{a}\left(\tau \right)<0$⇔$\left[\begin{array}{cc} -\Sigma_{i} & A_{L}^{T}\left(\varpi \left(\tau \right)\right)\\ A_{L}\left(\varpi \left(\tau \right)\right) & -\Sigma_{i}^{-1} \end{array}\right]<0$ is feasible if and only if $\left[\begin{array}{cc} -\Sigma_{i} & *\\ \Sigma_{i}A_{f_{i}}-\Lambda_{i}C & -\Sigma_{i} \end{array}\right]$ < 0, which is equivalent to (6) having a solution for $\Sigma_{i}\in R^{n}$ and $\Lambda_{i}\in R^{n\times p}$.    □

**Theorem** **1.** *Under Assumption 1, ${\tilde{x}}_{i}\left(0\right)\in \langle c_{i,0},R_{d}\left(\Theta_{i,0}\right)\rangle$. Moreover, we have*

(7) $$\begin{array}{c} \left\{\begin{array}{c} {\tilde{x}}_{i}\left(\tau \right)\in \langle c_{i,k},\Theta_{i,k}\rangle \subseteq \langle c_{i,k},R_{d}\left(\Theta_{i,k}\right)\rangle \\ c_{i,k}=A_{L}\left(\varpi \left(k-1\right)\right)c_{i,k-1}+B\left(\varpi \left(k-1\right)\right)g_{i,v}\\ \Theta_{i,k}=\left[\begin{array}{cc} A_{L}\left(\varpi \left(k-1\right)\right)R_{d}\left(\Theta_{i,k-1}\right) & B\left(\varpi \left(k-1\right)\right)G_{i,v} \end{array}\right] \end{array}\right. \end{array}$$

*Here, we denote $c_{i,0}=\frac{1}{2}\left({\bar{x}}_{i,0}+{\underline{x}}_{i,0}\right)-{\hat{x}}_{i,0},\Theta_{i,0}=\frac{1}{2}\mathrm{diag}\left({\bar{x}}_{i,0}-{\underline{x}}_{i,0}\right),g_{i,v}=\frac{1}{2}\left({\bar{v}}_{i}+{\underline{v}}_{i}\right)$, and $G_{i,v}=\frac{1}{2}\mathrm{diag}\left({\bar{v}}_{i}-{\underline{v}}_{i}\right)$. Furthermore, we define $R_{d}\left(\Theta_{i,0}\right)=\Theta_{i,0}$ and ${\hat{x}}_{i}\left(0\right)={\hat{x}}_{i,0}$.*

**Proof.**  Because of Assumption 1 and Property 4, we have $v_{i}\left(\tau \right)\in \langle g_{i,v},G_{i,v}\rangle$(k≥0). Based on (5) and using Property 1, one can proceed with the implementation of the subsequent iterative algorithm:

$$\begin{array}{c} \begin{array}{cc} \; & {\tilde{x}}_{i}\left(1\right)=A_{L}\left(\varpi \left(0\right)\right){\tilde{x}}_{i}\left(0\right)+B\left(\varpi \left(0\right)\right)v_{i}\left(0\right)\\ \; & \in \langle A_{L}\left(\varpi \left(0\right)\right)c_{i,0},A_{L}\left(\varpi \left(0\right)\right)\Theta_{i,0}\rangle ⊕\langle B\left(\varpi \left(0\right)\right)g_{i,v},B\left(\varpi \left(0\right)\right)G_{i,v}\rangle \\ \; & =\langle c_{i,1},\Theta_{i,1}\rangle \end{array} \end{array}$$

where $c_{i,1}=A_{L}\left(\varpi \left(0\right)\right)c_{i,0}+B\left(\varpi \left(0\right)\right)g_{i,v}$ and $\Theta_{i,1}=\left[A_{L}\left(\varpi \left(0\right)\right)\Theta_{i,0}B\left(\varpi \left(0\right)\right)G_{i,v}\right]$. To prevent the escalation of the zonotope’s order during the iterative algorithm, we can establish a fixed order d(n≤d) for the zonotope by utilizing Property 2.

$$\begin{array}{c} {\tilde{x}}_{i}\left(1\right)=\langle c_{i,1},\Theta_{i,1}\rangle \subseteq \langle c_{i,1},R_{d}\left(\Theta_{i,1}\right)\rangle \end{array}$$

where $R_{d}\left(\Theta_{i,1}\right)$ is defined in the same manner as $R_{d}\left(\Theta \right)$, as defined in Property 2. Furthermore,

$$\begin{array}{c} \begin{array}{cc} \; & {\tilde{x}}_{i}\left(2\right)=A_{L}\left(\varpi \left(1\right)\right){\tilde{x}}_{i}\left(1\right)+B\left(\varpi \left(1\right)\right)v_{i}\left(1\right)\\ \; & \in \langle A_{L}\left(\varpi \left(1\right)\right)c_{i,1},A_{L}\left(\varpi \left(1\right)\right)\Theta_{i,1}\rangle ⊕\langle B\left(\varpi \left(1\right)\right)g_{i,v},B\left(\varpi \left(1\right)\right)G_{i,v}\rangle \\ \; & =\langle c_{i,2},H_{i,2}\rangle \end{array} \end{array}$$

where $c_{i,2}=A_{L}\left(\varpi \left(1\right)\right)c_{i,1}+B\left(\varpi \left(1\right)\right)g_{i,v}$ and $\Theta_{i,2}=\left[A_{L}\left(\varpi \left(1\right)\right)\Theta_{i,1}\;B\left(\varpi \left(1\right)\right)G_{i,v}\right]$. Similarly, we can obtain the following conclusions based on Property 2:

$$\begin{array}{c} {\tilde{x}}_{i}\left(2\right)=\langle c_{i,2},\Theta_{i,2}\rangle \subseteq \langle c_{i,2},R_{d}\left(\Theta_{i,2}\right)\rangle \end{array}$$

In conclusion, we have established (7), which concludes the proof of Theorem 1.    □

Now, denote $R_{d}\left(\Theta_{i,k}\right)={\left[{\overset{˘}{\Theta }}_{i,k,1}^{T}\;\cdots \;{\overset{˘}{\Theta }}_{i,k,n}^{T}\right]}^{T}\in R^{n\times d},{\overset{˘}{\Theta }}_{i,k,r}=\left[\begin{array}{ccc} {\overset{˘}{\Theta }}_{i,k,r1} & \cdots & {\overset{˘}{\Theta }}_{i,k,rd} \end{array}\right]$$\in R^{1\times d}\left(r=1,\cdots ,n\right)$, and ${\hat{R}}_{d}\left(\Theta_{i,k}\right)={\left[\sum_{j=1}^{d}\left|{\overset{˘}{\Theta }}_{i,k,1j}\right|\cdots \sum_{j=1}^{d}\left|{\overset{˘}{\Theta }}_{i,k,nj}\right|\right]}^{T}$; then, according to (3) in Property 3, we have $c_{k}-{\hat{R}}_{d}\left(\Theta_{i,k}\right)\leq {\tilde{x}}_{i}\left(\tau \right)\leq c_{k}+{\hat{R}}_{d}\left(\Theta_{i,k}\right)$, which gives ${\underline{x}}_{i}\left(\tau \right)\leq x_{i}\left(\tau \right)\leq {\bar{x}}_{i}\left(\tau \right)$, where

(8) $$\begin{array}{c} \left\{\begin{array}{c} {\bar{x}}_{i}\left(\tau \right)=c_{k}+{\hat{x}}_{i}\left(\tau \right)+{\hat{R}}_{d}\left(\Theta_{i,k}\right)\\ {\underline{x}}_{i}\left(\tau \right)=c_{k}+{\hat{x}}_{i}\left(\tau \right)-{\hat{R}}_{d}\left(\Theta_{i,k}\right) \end{array}\right. \end{array}$$

with $c_{k}={\left[\begin{array}{ccc} c_{1,k} & \cdots & c_{n,k} \end{array}\right]}^{T}$. Moreover, notice that $y_{i}\left(\tau \right)=Cx_{i}\left(\tau \right)$; if we define

(9) $$\begin{array}{c} \left\{\begin{array}{c} {\bar{y}}_{i}\left(\tau \right)=C^{+}{\bar{x}}_{i}\left(\tau \right)-C^{-}{\underline{x}}_{i}\left(\tau \right)\\ {\underline{y}}_{i}\left(\tau \right)=C^{+}{\underline{x}}_{i}\left(\tau \right)-C^{-}{\bar{x}}_{i}\left(\tau \right) \end{array}\right. \end{array}$$

then, by Lemma 1, we have ${\underline{y}}_{i}\left(\tau \right)\leq y_{i}\left(\tau \right)\leq {\bar{y}}_{i}\left(\tau \right)$, which implies that there exists a meeting of $\beta_{i,j}\left(\tau \right)$ with $0\leq \beta_{i}\left(\tau \right)\leq 1_{n}$ such that

(10) $$\begin{array}{c} y_{i}\left(\tau \right)=\mathrm{diag}\left({\hat{y}}_{i}\left(\tau \right)\right)\beta_{i}\left(\tau \right)+{\underline{y}}_{i}\left(\tau \right) \end{array}$$

where ${\hat{y}}_{i}\left(\tau \right)={\left[\begin{array}{ccc} {\hat{y}}_{i,1}\left(\tau \right) & \cdots & {\hat{y}}_{i,p}\left(\tau \right) \end{array}\right]}^{T}$ and $\beta_{i}\left(\tau \right)={\left[\begin{array}{ccc} \beta_{i,1}\left(\tau \right) & \cdots & \beta_{i,p}\left(\tau \right) \end{array}\right]}^{T}$. Equation (10) gives

(11) $$\begin{array}{c} y_{i}\left(\tau +1\right)=\mathrm{diag}\left({\hat{y}}_{i}\left(\tau +1\right)\right)\beta_{i}\left(\tau +1\right)+{\underline{y}}_{i}\left(\tau +1\right) \end{array}$$

Based on (9), we obtain $\hat{y}\left(\tau +1\right)=\left|C\right|{\hat{x}}_{i}\left(\tau +1\right)$, where ${\hat{x}}_{i}\left(\tau +1\right)={\bar{x}}_{i}\left(\tau +1\right)$${\underline{x}}_{i}\left(\tau +1\right)$. It follows from (8) that ${\hat{x}}_{i}\left(\tau +1\right)=2{\hat{R}}_{d}\left(\Theta_{i,k+1}\right)$; thus, $\hat{y}\left(\tau +1\right)=2\left|C\right|{\hat{R}}_{d}\left(\Theta_{i,k+1}\right)$. Then, (11) becomes

(12) $$\begin{array}{cc} y_{i}\left(\tau +1\right) & =2\mathrm{diag}\left(\left|C\right|{\hat{R}}_{d}\left(\Theta_{i,k+1}\right)\right)\beta_{i}\left(\tau +1\right)+{\underline{y}}_{i}\left(\tau +1\right) \end{array}$$

According to the second equation in (9) and (8),

(13) $$\begin{array}{cc} \; & {\bar{y}}_{i}\left(\tau \right)=C\left(c_{k}+{\hat{x}}_{i}\left(\tau \right)\right)+\left|C\right|{\hat{R}}_{d}\left(\Theta_{i,k}\right)\\ \; & {\underline{y}}_{i}\left(\tau \right)=C\left(c_{k}+{\hat{x}}_{i}\left(\tau \right)\right)-\left|C\right|{\hat{R}}_{d}\left(\Theta_{i,k}\right) \end{array}$$

Based on (13) and (12), we have

(14) $$\begin{array}{c} y_{i}\left(\tau +1\right)=2\mathrm{diag}\left(\left|C\right|{\hat{R}}_{d}\left(\Theta_{i,k+1}\right)\right)\beta_{i}\left(\tau +1\right)+C\left(c_{k+1}+{\hat{x}}_{i}\left(\tau +1\right)\right)-\left|C\right|{\hat{R}}_{d}\left(\Theta_{i,k+1}\right) \end{array}$$

Using (14) and (4), we can deduce that

(15) $$\begin{array}{c} y_{i}\left(\tau +1\right)=2\mathrm{diag}\left(\left|C\right|{\hat{R}}_{d}\left(\Theta_{i,k+1}\right)\right)\beta_{i}\left(\tau +1\right)+\phi \left(\tau \right)+CB\left(\varpi \left(\tau \right)\right)u_{i}\left(\tau \right) \end{array}$$

where $\phi \left(\tau \right)=CA_{L}\left(\varpi \left(\tau \right)\right){\hat{x}}_{i}\left(\tau \right)+CL\left(\varpi \left(\tau \right)\right)y\left(\tau \right)+Cc_{k+1}-\left|C\right|{\hat{R}}_{d}\left(\Theta_{i,k+1}\right)$. On the other hand, from (1), we have

(16) $$\begin{array}{c} y_{i}\left(\tau +1\right)=CA\left(\varpi \left(\tau \right)\right)x_{i}\left(\tau \right)+CB\left(\varpi \left(\tau \right)\right)u_{i}\left(\tau \right)+CB\left(\varpi \left(\tau \right)\right)v_{i}\left(\tau \right) \end{array}$$

Compare (15) with (16); then, we can obtain that, by using the Plant Rule in Section 2.3,

$$\begin{array}{c} CB_{f_{i}}v_{i}\left(\tau \right)=2\mathrm{diag}\left(\left|C\right|{\hat{R}}_{d}\left(\Theta_{i,k+1}\right)\right)\beta_{i}\left(\tau +1\right)+\phi \left(\tau \right)-CA_{f_{i}}x_{i}\left(\tau \right) \end{array}$$

or, under Assumption 2,

$$\begin{array}{c} v_{i}\left(\tau \right)={\left(CB_{f_{i}}\right)}^{†}\left[2\mathrm{diag}\left(\left|C\right|{\hat{R}}_{d}\left(\Theta_{i,k+1}\right)\right)\beta_{i}\left(\tau +1\right)+\phi \left(\tau \right)-CA_{f_{i}}x_{i}\left(\tau \right)\right] \end{array}$$

where ${\left(CB_{f_{i}}\right)}^{†}={\left[{\left(CB_{f_{i}}\right)}^{T}CB_{f_{i}}\right]}^{-1}{\left(CB_{f_{i}}\right)}^{T}$. Then, based on the fuzzy inference through the center-average defuzzifier, we have

(17) $$\begin{array}{c} v_{i}\left(\tau \right)=\Gamma \left(\varpi \left(\tau \right)\right)\left[2\mathrm{diag}\left(\left|C\right|{\hat{R}}_{d}\left(\Theta_{i,k+1}\right)\right)\beta_{i}\left(\tau +1\right)+\phi \left(\tau \right)-CA\left(\varpi \left(\tau \right)\right)x_{i}\left(\tau \right)\right] \end{array}$$

where $\Gamma \left(\varpi \left(\tau \right)\right)=\sum_{f_{i}=1}^{l}\varpi_{f_{i}}\left(w_{i}\left(\tau \right)\right){\left(CB_{f_{i}}\right)}^{†}$. As follows, the computation of $\beta_{i}\left(\tau \right)$ can be given. Exactly, based on (11), we have

(18) $$\begin{array}{cc} \beta_{i}\left(\tau +1\right)\; & =\left\{{\left[\mathrm{diag}\left(2|C|{\hat{R}}_{d}\left(\Theta_{i,k+1}\right)+\theta_{i}\left(\tau \right)\right)\right]}^{-1}-\mathrm{diag}\left(\theta_{i}\left(\tau \right)\right)\right\}\\ \; & \times \left[y_{i}\left(\tau +1\right)-{\underline{y}}_{i}\left(\tau +1\right)\right] \end{array}$$

where ${\underline{y}}_{i}\left(\tau +1\right)=\phi \left(\tau \right)+CB\left(\varpi \left(\tau \right)\right)u_{i}\left(\tau \right)$. Here, $\theta_{i}\left(\tau \right)={\left[\begin{array}{ccc} \theta_{i,1}\left(\tau \right) & \cdots & \theta_{i,p}\left(\tau \right) \end{array}\right]}^{T}$$\theta_{i,l}\left(\tau \right)=1$ if the *l*th element of $\left|C\right|{\hat{R}}_{d}\left(\Theta_{i,k+1}\right)$ is zeros; otherwise, $\theta_{i,l}\left(\tau \right)=0$(l=1,⋯,p).

Now, using (18), we design a UIO

(19) $$\begin{array}{c} \left\{\begin{array}{c} \xi_{i}\left(\tau +1\right)=A\left(\varpi \left(\tau \right)\right)\xi_{i}\left(\tau \right)+B\left(\varpi \left(\tau \right)\right)u_{i}\left(\tau \right)+B\left(\varpi \left(\tau \right)\right){\hat{v}}_{i}\left(\tau \right)\\ +M\left(\varpi \left(\tau \right)\right)\left(y_{i}\left(\tau \right)-C\xi_{i}\left(\tau \right)\right)\\ {\hat{v}}_{i}\left(\tau \right)=\Gamma \left(\varpi \left(\tau \right)\right)\left[2\mathrm{diag}\left(\left|C\right|{\hat{R}}_{d}\left(\Theta_{i,k+1}\right)\right)\beta_{i}\left(\tau +1\right)+\phi \left(\tau \right)-CA\left(\varpi \left(\tau \right)\right)\xi_{i}\left(\tau \right)\right] \end{array}\right. \end{array}$$

where $M\left(\varpi \left(\tau \right)\right)=\sum_{f_{i}=1}^{l}\varpi_{f_{i}}\left(w_{i}\left(\tau \right)\right)M_{f_{i}}$ and $M_{f_{i}}\in R^{p\times n}$ is the matrix gain to be designed.

**Remark** **2.** *It should be emphasized that it is difficult for one to obtain the state interval estimation by designing a traditional interval observer for a T–S fuzzy system. Therefore, a combination of the zonotope theory and a Luenberger observer plays a critical role in addressing the interval estimation problem for T–S fuzzy models. Specifically, the Luenberger-like observer provides a rough state estimation because of the existence of the external disturbance. And then, based on the error dynamic Luenberger-like observer system together with the zonotope theory, an algebraic iterative calculation for state interval estimation can be put forward. In addition, by using the state interval estimates, an algebraic relationship between the external disturbance and the system state is established. Moreover, by using the algebraic relationship, a UIO that consists of a Luenberger-like state observer and an unknown input reconstruction is developed. And the UIO is able to asymptotically estimate the system state and the external disturbance simultaneously. In this way, a new theoretical framework of UIO through the combination of the zonotope theory and the Luenberger observer techniques is set up.*

**Theorem** **2.** *With Assumptions 1–3, (19) is a UIO of (1), being able to estimate x and v asymptotically.*

**Proof.**  To begin with, subtracting the first equation in (19) from (1) gives

(20) $$\begin{array}{c} {\tilde{\xi }}_{i}\left(\tau +1\right)=A_{M}\left(\varpi \left(\tau \right)\right){\tilde{\xi }}_{i}\left(\tau \right)+B\left(\varpi \left(\tau \right)\right){\tilde{v}}_{i}\left(\tau \right) \end{array}$$

where $A_{M}\left(\varpi \left(\tau \right)\right)=A\left(\varpi \left(\tau \right)\right)-M\left(\varpi \left(\tau \right)\right)C=\sum_{f_{i}=1}^{l}\varpi_{f_{i}}\left(w_{i}\left(\tau \right)\right)\left(A_{f_{i}}-M_{f_{i}}C\right)$. Additionally, subtracting the second equation in (19) from (17) yields

(21) $$\begin{array}{c} {\tilde{v}}_{i}\left(\tau \right)=-\Gamma \left(\varpi \left(\tau \right)\right)CA\left(\varpi \left(\tau \right)\right){\tilde{\xi }}_{i}\left(\tau \right) \end{array}$$

Moreover, substitute (21) into (20) to obtain

(22) $$\begin{array}{cc} {\tilde{\xi }}_{i}\left(\tau +1\right) & =\left(A_{M}\left(\varpi \left(\tau \right)\right)-B\left(\varpi \left(\tau \right)\right)\Gamma \left(\varpi \left(\tau \right)\right)CA\left(\varpi \left(\tau \right)\right)\right){\tilde{\xi }}_{i}\left(\tau \right) \end{array}$$

The overall system of (22) is

(23) $$\begin{array}{cc} \tilde{\xi }\left(\tau +1\right) & =\left[I_{N}⊗\left(A_{M}\left(\varpi \left(\tau \right)\right)-B\left(\varpi \left(\tau \right)\right)\Gamma \left(\varpi \left(\tau \right)\right)CA\left(\varpi \left(\tau \right)\right)\right)\right]\tilde{\xi }\left(\tau \right) \end{array}$$

where $\tilde{\xi }\left(\tau \right)={\left[\begin{array}{ccc} {\tilde{\xi }}_{1}^{T}\left(\tau \right) & \ldots & {\tilde{\xi }}_{N}^{T}\left(\tau \right) \end{array}\right]}^{T}$.   □

**Remark** **3.** *It is noted that (17) establishes a relationship of the UI and the state algebraically and it does not involve the control input $u_{i}\left(\tau \right)$. Using this correlation, a UIO being composed of a Luenberger-like observer and a UIR is formulated and is expressed by (19). The proposed UIO has several advantages. Firstly, it can give the asymptotic estimations of both the state and UI simultaneously. Secondly, the control input $u_{i}\left(\tau \right)$ is decoupled from the UIR. Thirdly, the asymptotic convergence property is unaffected by the ${\bar{v}}_{i}$ and ${\underline{v}}_{i}$, which makes Assumption 1 be very relaxed.*

## 4. UIO-Based Distributed Consensus Control

We propose a distributed protocol based on UIO to accomplish the T–S fuzzy MAS consensus illustrated by Definition 2 in this section. And both the gains of the UIO and the controller are determined by LMIs.

Firstly, define $\chi_{i}\left(\tau \right)=x_{i}\left(\tau \right)-x_{0}\left(\tau \right)$, and then its dynamic system can be set up as

(24) $$\begin{array}{c} \chi_{i}\left(\tau +1\right)=A\left(\varpi \left(\tau \right)\right)\chi_{i}\left(\tau \right)+B\left(\varpi \left(\tau \right)\right)\left(u_{i}\left(\tau \right)+v_{i}\left(\tau \right)\right) \end{array}$$

Now, for (24), a UIO-based distributed controller is built:

(25) $$\begin{array}{c} u_{i}\left(\tau \right)=-\alpha K\left(\varpi \left(\tau \right)\right)\left[\sum_{j=1}^{N}a_{ij}\left(\xi_{i}\left(\tau \right)-\xi_{j}\left(\tau \right)\right)+b_{i}\left(\xi_{i}\left(\tau \right)-x_{0}\left(\tau \right)\right)\right]-{\hat{v}}_{i}\left(\tau \right) \end{array}$$

where $K\left(\varpi \left(\tau \right)\right)=\sum_{f_{i}=1}^{l}\varpi_{f_{i}}\left(w_{i}\left(\tau \right)\right)K_{f_{i}}$ and $K_{f_{i}}\in R^{m\times n}$ is the state feedback gain matrix to be designed. Now, using (25), the closed-loop dynamic system of (24) is

$$\begin{array}{cc} \chi_{i}\left(\tau +1\right) & =A\left(\varpi \left(\tau \right)\right)\chi_{i}\left(\tau \right)-\alpha B\left(\varpi \left(\tau \right)\right)K\left(\varpi \left(w\left(\tau \right)\right)\right)\left[\sum_{j=1}^{N}a_{ij}\left(\chi_{i}\left(\tau \right)-\chi_{j}\left(\tau \right)\right)+b_{i}\chi_{i}\left(\tau \right)\right]\\ \; & +\alpha B\left(\varpi \left(\tau \right)\right)K\left(\varpi \left(w\left(\tau \right)\right)\right)\left[\sum_{j=1}^{N}a_{ij}\left({\tilde{\xi }}_{i}\left(\tau \right)-{\tilde{\xi }}_{j}\left(\tau \right)\right)+b_{i}{\tilde{\xi }}_{i}\left(\tau \right)\right]+B\left(\varpi \left(\tau \right)\right){\tilde{v}}_{i}\left(\tau \right) \end{array}$$

Next, substituting (21) into above equation yields

(26) $$\begin{array}{cc} \chi_{i}\left(\tau +1\right) & =A\left(\varpi \left(\tau \right)\right)\chi_{i}\left(\tau \right)-\alpha B\left(\varpi \left(\tau \right)\right)K\left(\varpi \left(w\left(\tau \right)\right)\right)\left[\sum_{j=1}^{N}a_{ij}\left(\chi_{i}\left(\tau \right)-\chi_{j}\left(\tau \right)\right)+b_{i}\chi_{i}\left(\tau \right)\right]\\ \; & +\alpha B\left(\varpi \left(\tau \right)\right)K\left(\varpi \left(w\left(\tau \right)\right)\right)\left[\sum_{j=1}^{N}a_{ij}\left({\tilde{\xi }}_{i}\left(\tau \right)-{\tilde{\xi }}_{j}\left(\tau \right)\right)+b_{i}{\tilde{\xi }}_{i}\left(\tau \right)\right]\\ \; & -B\left(\varpi \left(\tau \right)\right)\Gamma \left(\varpi \left(\tau \right)\right)CA\left(\varpi \left(\tau \right)\right){\tilde{\xi }}_{i}\left(\tau \right) \end{array}$$

The overall system of (26) is

(27) $$\begin{array}{cc} \chi (\tau +1) & =\left[I_{N}⊗A\left(\varpi \left(\tau \right)\right)-\alpha H⊗B\left(\varpi \left(\tau \right)\right)K\left(\varpi \left(\tau \right)\right)\right]\chi \left(\tau \right)+[\alpha \left(H⊗B\left(\varpi \left(\tau \right)\right)K\left(\varpi \left(\tau \right)\right)\right)\\ \; & -I_{N}⊗\left(B\left(\varpi \left(\tau \right)\right)\Gamma \left(\varpi \left(\tau \right)\right)CA\left(\varpi \left(\tau \right)\right)\right)]\tilde{\xi }\left(\tau \right) \end{array}$$

where $\chi \left(\tau \right)={\left[\begin{array}{ccc} \chi_{1}^{T}\left(\tau \right) & \ldots & \chi_{N}^{T}\left(\tau \right) \end{array}\right]}^{T}$. Define $\psi \left(\tau \right)={\left[\begin{array}{cc} \chi^{T}\left(\tau \right) & \xi^{T}\left(\tau \right) \end{array}\right]}^{T}$ and combine (27) and (23); then, we can obtain

(28) $$\begin{array}{c} \psi (\tau +1)=\Omega (\varpi (\tau ))\psi (\tau ) \end{array}$$

where

(29) $$\begin{array}{c} \Omega \left(\varpi \left(\tau \right)\right)=\left[\begin{array}{cc} I_{N}⊗A-\alpha H⊗BK & \alpha \left(H⊗BK\right)-I_{N}⊗\left(B\Gamma CA\right)\\ 0 & I_{N}⊗\left(A_{M}-B\Gamma CA\right) \end{array}\right] \end{array}$$

It should be emphasized that (29) is expressed in a brief form, in that all A(ϖ(τ)), $A_{M}\left(\varpi \left(\tau \right)\right),B\left(\varpi \left(\tau \right)\right),K\left(\varpi \left(\tau \right)\right)$, and Γ(ϖ(τ)) are written in brief as $A,A_{M},B,K$, and Γ.

**Theorem** **3.** *If there are $P_{1}>0$ and $P_{2}>0$, and matrix $X_{f_{i}}$ satisfying LMIs*

(30) $$\begin{array}{c} \left[\begin{array}{cc} \Xi_{1,f_{i},f_{j},f_{k}} & *\\ \Xi_{2,f_{i}} & -I_{2Nm} \end{array}\right]<0 \end{array}$$

*where*

$$\begin{array}{c} \Xi_{1,f_{i},f_{j},f_{k}}=\left[\begin{array}{cccc} -I_{N}⊗P_{1} & * & * & *\\ 0 & -I_{N}⊗P_{2} & * & *\\ I_{N}⊗P_{1}A_{f_{i}} & -I_{N}⊗\Xi_{3,f_{i},f_{j},f_{k}} & -I_{N}⊗P_{1} & *\\ 0 & I_{N}⊗\Xi_{4,f_{i},f_{j},f_{k}} & 0 & -I_{N}⊗P_{2} \end{array}\right], \end{array}$$

*$\Xi_{3,f_{i},f_{j},f_{k}}=\left(P_{1}B_{f_{i}}{\left(CB_{f_{j}}\right)}^{†}CA_{f_{k}}\right)$, then $\Xi_{4,f_{i},f_{j},f_{k}}=\left(P_{2}\left(A_{f_{i}}-M_{f_{i}}C\right)-P_{2}B_{f_{i}}{\left(CB_{f_{j}}\right)}^{†}CA_{f_{k}}\right)$ and $\Xi_{2,f_{i}}=\left[\begin{array}{cccc} 0 & 0 & 0 & 0\\ \sqrt{\alpha H}⊗B_{f_{i}}^{T}P & 0 & \sqrt{\alpha H}⊗B_{f_{i}}^{T}P_{1} & 0 \end{array}\right]$. $\left(f_{i},f_{j},f_{k}=1,\cdots ,l\right)$ is feasible; then, under (25), MASs (1) and (2) can fulfill the asymptotic convergence consensus.*

**Proof.**  Consider Lyapunov function candidate $V\left(\tau \right)=\chi^{T}\left(\tau \right)\left(I_{N}⊗P_{1}\right)\chi \left(\tau \right)+{\tilde{\xi }}^{T}\left(\tau \right)\left(I_{N}⊗P_{2}\right)\tilde{\xi }\left(\tau \right)=\psi^{T}\left(\tau \right)P\psi \left(\tau \right),$ where $P_{1}>0$ and $P_{2}>0,P=\left[\begin{array}{cc} I_{N}⊗P_{1} & 0\\ 0 & I_{N}⊗P_{2} \end{array}\right]$. And the difference of V(τ) along with the trajectory of the error dynamic system (28) is

$$\begin{array}{cc} \Delta V(\tau ) & =V(\tau +1)-V(\tau )\\ \; & =\psi^{T}\left(\tau \right)\left[\Omega^{T}\left(\varpi \left(\tau \right)\right)P\Omega \left(\varpi \left(\tau \right)\right)-P\right]\psi \left(\tau \right) \end{array}$$

By Schur’s complement lemma, we know that $\Omega^{T}\left(\varpi \left(\tau \right)\right)P\Omega \left(\varpi \left(\tau \right)\right)-P<0$ if and only if

$$\begin{array}{cc} \; & \left[\begin{array}{cc} -P & *\\ \Omega & -P^{-1} \end{array}\right]\\ \; & =\left[\begin{array}{cccc} -I_{N}⊗P_{1} & * & * & *\\ 0 & -I_{N}⊗P_{2} & * & *\\ I_{N}⊗A-\alpha H⊗BK & \alpha \left(H⊗BK\right)-I_{N}⊗\left(B\Gamma CA\right) & -I_{N}⊗P_{1}^{-1} & *\\ 0 & I_{N}⊗\left(A_{M}-B\Gamma CA\right) & 0 & -I_{N}⊗P_{2}^{-1} \end{array}\right]<0 \end{array}$$

if and only if

$$\begin{array}{c} \left[\begin{array}{cccc} -I_{N}⊗P_{1} & * & * & *\\ 0 & -I_{N}⊗P_{2} & * & *\\ I_{N}⊗P_{1}A-\alpha H⊗P_{1}BK & \alpha \left(H⊗P_{1}BK\right)-I_{N}⊗\left(P_{1}B\Gamma CA\right) & -I_{N}⊗P_{1} & *\\ 0 & I_{N}⊗\left(P_{2}A_{M}-P_{2}B\Gamma CA\right) & 0 & -I_{N}⊗P_{2} \end{array}\right]<0 \end{array}$$

and this is equivalent to, by setting $K=-B^{T}P_{1}$,

(31) $$\begin{array}{cc} \; & \left[\begin{array}{cccc} -I_{N}⊗P_{1} & * & * & *\\ 0 & -I_{N}⊗P_{2} & * & *\\ I_{N}⊗P_{1}A & -I_{N}⊗\left(P_{1}B\Gamma CA\right) & -I_{N}⊗P_{1} & *\\ 0 & I_{N}⊗\left(P_{2}A_{M}-P_{2}B\Gamma CA\right) & 0 & -I_{N}⊗P_{2} \end{array}\right]\\ \; & +\left[\begin{array}{cccc} \alpha H⊗P_{1}BB^{T}P_{1} & * & * & *\\ 0 & 0 & * & *\\ \alpha H⊗P_{1}BB^{T}P_{1} & 0 & \alpha H⊗P_{1}BB^{T}P_{1} & *\\ 0 & 0 & 0 & 0 \end{array}\right]-\left[\begin{array}{cccc} 0 & * & * & *\\ 0 & 0 & * & *\\ 0 & \alpha H⊗P_{1}BB^{T}P_{1} & 0 & *\\ 0 & 0 & 0 & 0 \end{array}\right]\\ \; & -\left[\begin{array}{cccc} \alpha H⊗P_{1}BB^{T}P_{1} & * & * & *\\ 0 & 0 & * & *\\ 0 & 0 & \alpha H⊗P_{1}BB^{T}P_{1} & *\\ 0 & 0 & 0 & 0 \end{array}\right]\\ \; & \leq \left[\begin{array}{cccc} -I_{N}⊗P_{1} & * & * & *\\ 0 & -I_{N}⊗P_{2} & * & *\\ I_{N}⊗P_{1}A & \alpha (H⊗BK) & -I_{N}⊗P_{1} & *\\ 0 & I_{N}⊗\left(P_{2}A_{M}-P_{2}B\Gamma CA\right) & 0 & -I_{N}⊗P_{2} \end{array}\right]+\Xi_{2}^{T}\Xi_{2}\\ \; & =\left[\begin{array}{cc} \Xi_{1} & \Xi_{2}^{T}\\ \Xi_{2} & -I_{2Nm} \end{array}\right]<0 \end{array}$$

where

$$\begin{array}{c} \Xi_{1}=\left[\begin{array}{cccc} -I_{N}⊗P_{1} & * & * & *\\ 0 & -I_{N}⊗P_{2} & * & *\\ I_{N}⊗P_{1}A & \alpha (H⊗BK) & * & *\\ 0 & I_{N}⊗\left(P_{2}A_{M}-P_{2}B\Gamma CA\right) & 0 & -I_{N}⊗P_{2} \end{array}\right] \end{array}$$

and $\Xi_{2}=\left[\begin{array}{cccc} 0 & 0 & 0 & 0\\ \sqrt{\alpha H}⊗P_{1}B & 0 & \sqrt{\alpha H}⊗B^{T}P_{1} & 0 \end{array}\right]$. And then (31) can be rewritten as

$$\begin{array}{cc} \; & \sum_{f_{i}=1}^{N}\sum_{f_{j}=1}^{N}\sum_{f_{k}=1}^{N}\left(\varpi_{f_{i}}\varpi_{f_{j}}\varpi_{f_{k}}\right)\left[\begin{array}{cc} \Xi_{1,f_{i},f_{j},f_{k}} & *\\ \Xi_{2,f_{i}} & -I_{2Nm} \end{array}\right]<0 \end{array}$$

which is equivalent to

$$\begin{array}{c} \left[\begin{array}{cc} \Xi_{1,f_{i},f_{j},f_{k}} & *\\ \Xi_{2,f_{i}} & -I_{2Nm} \end{array}\right]<0. \end{array}$$

   □

The Algorithm 1 offers the way of finding the solutions of symmetric positive definite matrices $P_{1}$ and $P_{2}$ and matrix $X_{f_{i}}$ such that (30) holds. Then, the calculations of the gain matrices of $M_{f_{i}}$ can be carried out. Then, one has $\lim_{\tau \to \infty }\Delta V\left(\tau \right)\leq 0$. And $\lim_{\tau \to \infty }\Delta V\left(\tau \right)=0$ if and only if $\lim_{\tau \to \infty }\psi \left(\tau \right)=0$. Thus, $\lim_{\tau \to \infty }\Delta V\left(\tau \right)=0$ means that $\lim_{\tau \to \infty }\psi \left(\tau \right)=0$.
**Algorithm 1** The calculation method of $u_{i}$**Require** system matrices $A_{f_{i}},B_{f_{i}},C\left(f_{i}=1,\cdots ,l\right)$, scalar α.
Step 1. Select appropriate $K_{f_{i}},\left(f_{i}=1,\cdots ,l\right)$ through pole placement method;
Step 2. Then (30) is LMIs, and solve (30) to obtain $P_{1}>0,P_{2}>0$ and $X_{f_{i}};$
Step 3. If there is no solution for (30), then return to Step 1;
Step 4. Set $M_{f_{i}}=P_{2}^{-1}X_{f_{i}}$;
Step 5. Design the distributed controller $u_{i}$ according to Equation (28).

**Remark** **4.** *The proposed UIO-based distributed control protocol in (25) exhibits significant advantages, particularly in the compensation for unknown input $v_{i}\left(\tau \right)$ by introducing its estimation into the controller. This compensation capability is attributed by the novel unknown input reconstruction (the second equation in (19)) with the feature of being decoupled from the control input. Consequently, under the UIO-based distributed control protocol in (24), the asymptotic convergence MAS consensus is fulfilled, and the superiority of this approach is evident by comparing it with existing methods, showing that our method has strong ability in dealing with unknown disturbances.*

## 5. Simulations

**Figure 1:**
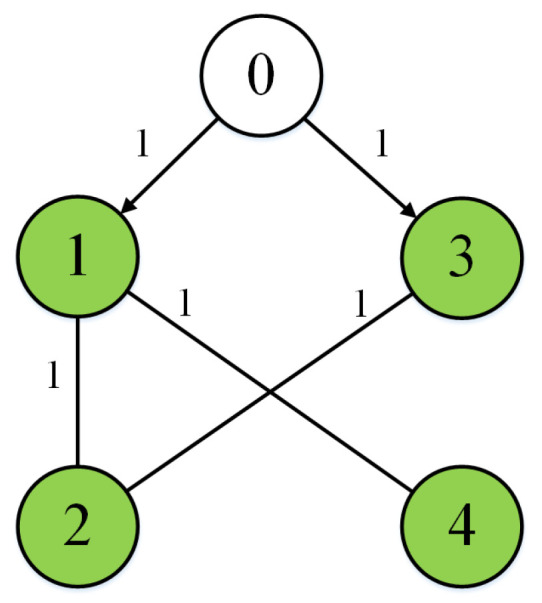
Communication graph.

Two simulation examples, in which one is a numerical example and the other is given based on a practical system, are provided to test the effectiveness of the proposed method in this section. Consider a T–S fuzzy MAS containing one leader and four followers. The communication graph is shown in Figure 1.

**Example** **1.** *A numerical example*

*The matrices for the dynamics of each agent are*

$$\begin{array}{c} \begin{array}{c} A_{1}=\left[\begin{array}{ccc} -0.9 & -1 & 0\\ 0 & 0.35 & 0\\ -1 & 1 & -0.24 \end{array}\right],A_{2}=\left[\begin{array}{ccc} -0.7 & -1 & 0\\ 0 & 0.65 & 0\\ -1 & -1 & -0.24 \end{array}\right],\\ B_{1}=\left[\begin{array}{c} 0\\ 0.1\\ 0.2 \end{array}\right],B_{2}=\left[\begin{array}{c} 0\\ -0.1\\ -0.2 \end{array}\right],C=\left[\begin{array}{ccc} 1 & 0 & 1\\ 1 & 1 & 0 \end{array}\right]. \end{array} \end{array}$$

*We determine fuzzy weights $\varpi_{1}\left(x_{3}\left(\tau \right)\right)=\frac{1-1/\left(\exp\left(-14\left(x_{3}\left(\tau \right)-\pi /8\right)\right)+1\right)}{\exp\left(-14\left(x_{3}\left(\tau \right)+\pi /8\right)\right)+1}$, and $\varpi_{2}\left(x_{3}\right)=1-\varpi_{1}\left(x_{3}\left(\tau \right)\right)$. In this example, the unknown inputs are supposed to be $v_{1}\left(\tau \right)=0.5\sin\left(0.5\tau \right)$, $v_{2}\left(\tau \right)=0.2\cos\left(0.5\tau \right)+1$, $v_{3}\left(\tau \right)=0.4\sin\left(0.5\tau +2\right)$, and $v_{4}\left(\tau \right)=0.4\cos\left(0.5\tau +2\right)$.*

The parameters in LMI (30) are selected as α=0.5. Select appropriate $K_{1}$ and $K_{2}$:

$$\begin{array}{c} \begin{array}{c} K1=\left[\begin{array}{ccc} -0.4372 & -0.4344 & 0.0185 \end{array}\right],\;K2=\left[\begin{array}{ccc} 0.4460 & 0.4412 & -0.0378 \end{array}\right]. \end{array} \end{array}$$

Then, by LMI (30), we can obtain $P_{1}$, $P_{2}$, $X_{1}$, and $X_{2}$:

$$\begin{array}{c} \begin{array}{c} P_{1}=\left[\begin{array}{ccc} 80.3730 & 55.4259 & -15.7023\\ 55.4259 & 132.4242 & -19.3739\\ -15.7023 & -19.3739 & 15.8545 \end{array}\right],P_{2}=\left[\begin{array}{ccc} 244.9000 & 53.3857 & 7.5050\\ 53.3857 & 206.8203 & 11.9223\\ 7.5050 & 11.9223 & 148.1690 \end{array}\right],\\ X_{1}=\left[\begin{array}{cc} 21.4569 & -191.4806\\ 50.1327 & 94.8898\\ -23.8080 & 165.3690 \end{array}\right],X_{2}=\left[\begin{array}{cc} 13.9241 & -149.4792\\ -25.6183 & 227.3682\\ -10.4426 & 116.0064 \end{array}\right]. \end{array} \end{array}$$

and we can calculate $M_{1}$ and $M_{2}$ as

$$\begin{array}{c} \begin{array}{c} M_{1}=\left[\begin{array}{cc} 0.0403 & -0.9558\\ 0.2425 & 0.6414\\ -0.1822 & 1.1129 \end{array}\right],M_{2}=\left[\begin{array}{cc} 0.0901 & -0.9146\\ -0.1435 & 1.2936\\ -0.0635 & 0.7252 \end{array}\right]. \end{array} \end{array}$$

**Figure 2:**
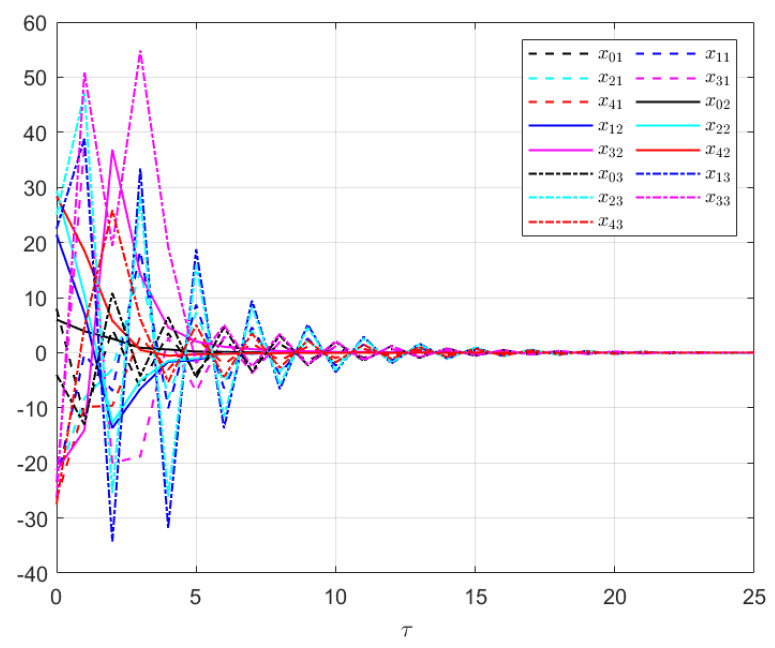
The UIO−based distributed control consensus performance in Example 1.

**Figure 3:**
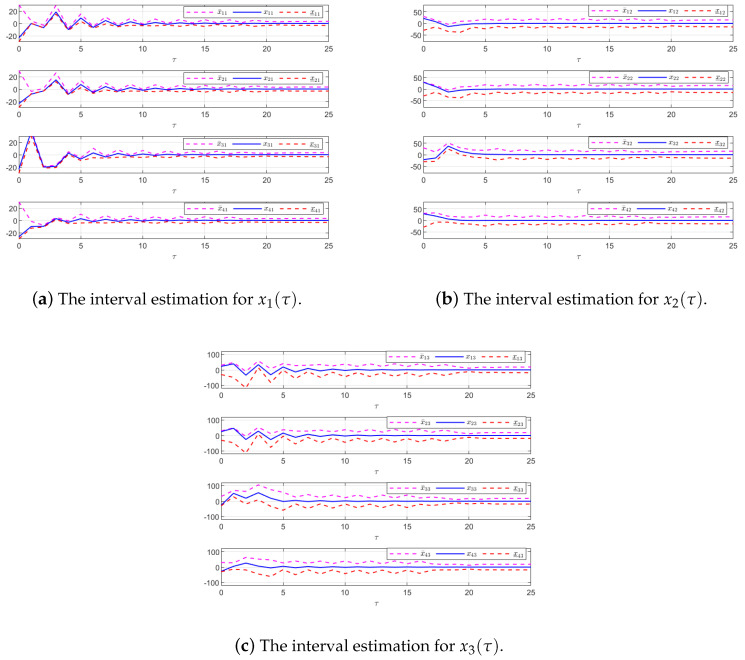
The interval estimation performance in Example 1.

**Figure 4:**
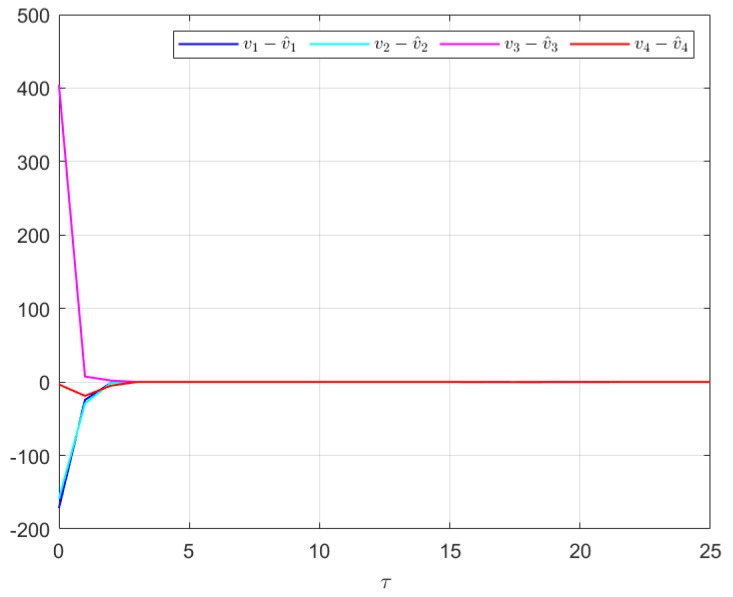
The UIR performance in Example 1.

**Figure 5:**
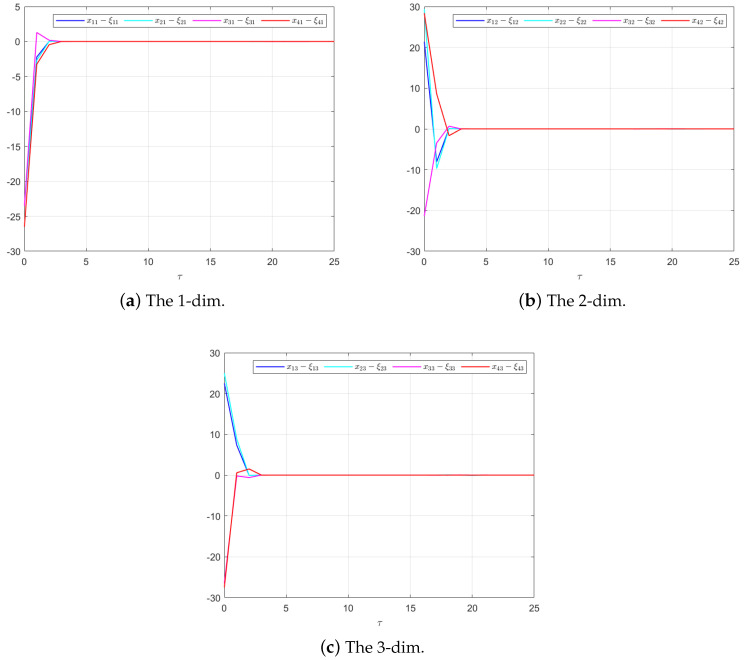
The state estimation performances for UIO (19) in Example 1.

**Figure 6:**
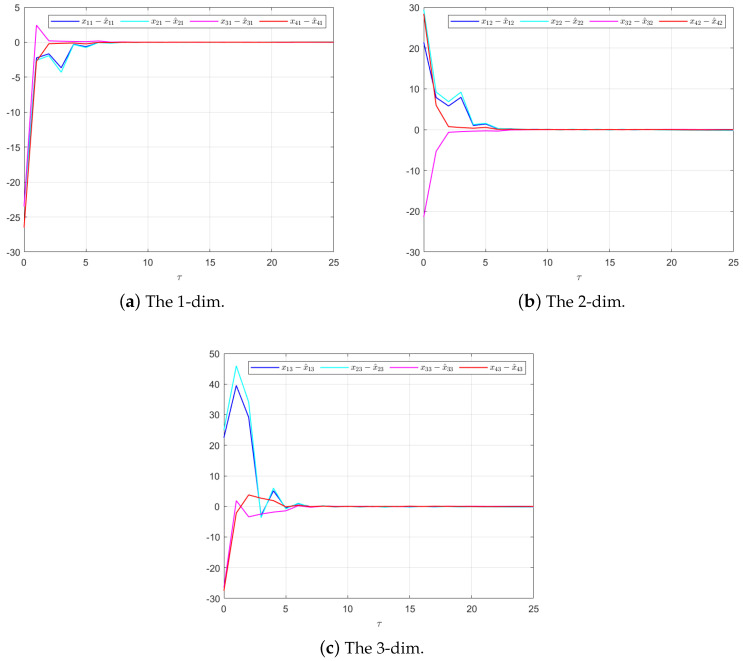
The state estimation performances for Luenberger-like Observer (4) in Example 1.

The effectiveness of the UIO-based distributed controller in (25) can be verified by Figure 2, Figure 3, Figure 4, Figure 5 and Figure 6. Each figure highlights different aspects of the system’s performance and validates the proposed control protocol. In Figure 2, the trajectories of the states for each follower and the leader are plotted. It is evident that, under the proposed control protocol, consensus is achieved asymptotically. This is particularly significant in the context of T–S fuzzy MASs, where achieving consensus despite uncertainties and external disturbances can be challenging. The alignment of state trajectories in the figure indicates that all the followers successfully converge to the leader’s state over time, verifying the controller’s effectiveness in achieving the desired cooperative behavior. The alignment of state trajectories indicates that all followers are able to converge to the leader’s state asymptotically. Figure 3 presents both the interval estimates and the actual states. The interval estimates effectively capture and enclose the actual state trajectories, confirming that the proposed controller provides reliable state estimation within bounded intervals. In Figure 4, the reconstruction of the UI is demonstrated. The figure shows that the UI can be accurately and asymptotically reconstructed using the proposed UIO framework, as governed by the second equation in (19). Figure 5 and Figure 6 provide a detailed view of the state estimation performance over time. These figures illustrate the asymptotic convergence of the state estimates, demonstrating the accuracy and stability of the proposed control scheme.

**Example** **2.** *A practical system*

*Similar to [25], consider Duffing–Van der Pol’s oscillator model with the following dynamics:*

$$\begin{array}{c} {\ddot{m}}_{i}\left(t\right)+\omega^{2}m_{i}\left(t\right)-\left(\alpha -\beta m_{i}^{2}\left(t\right)\right){\dot{m}}_{i}\left(t\right)+\gamma m_{i}^{3}\left(t\right)=u_{i}\left(t\right)+v_{i}\left(t\right), \end{array}$$

*where $m_{i}\left(t\right)$, $u_{i}\left(t\right)$, and $v_{i}\left(t\right)$ denote the position, control input, and external disturbance of the ith oscillator. γ denotes the coefficient of the nonlinear stiffness. ω represents the natural frequency. α and β are the damping strengths.*

Define $x_{i}\left(t\right)={\left[m_{i}\left(t\right)\;{\dot{m}}_{i}\left(t\right)\right]}^{T}$ and compact set $D=\{|m_{i}\left(t\right)|\leq a_{1},|{\dot{m}}_{i}\left(t\right)|\leq a_{2}\}$, where $a_{1}$ and $a_{2}$ are two positive constants. Then, the above nonlinear system can be reformulated as the subsequent fuzzy model.

**Plant Rule** $f_{i}$: IF $w_{i1}\left(\tau \right)$ is $F_{f_{i}1}$, THEN

(32) $$\begin{array}{c} {\dot{x}}_{i}\left(t\right)=A_{f_{i}}x_{i}\left(t\right)+B_{f_{i}}\left(u_{i}\left(t\right)+v_{i}\left(t\right)\right), \end{array}$$

where

$$\begin{array}{c} A_{1}=\left[\begin{array}{cc} 0 & 1\\ -\omega^{2}-\gamma *a_{1}^{2} & \alpha -\beta *a_{1}^{2} \end{array}\right],\;A_{2}=\left[\begin{array}{cc} 0 & 1\\ -\omega^{2} & \alpha \end{array}\right],\;B_{1}=B_{2}=\left[\begin{array}{c} 0\\ 1 \end{array}\right] \end{array}$$

with $\omega =\sqrt{2},\alpha =0.1,\beta =0.05,\gamma =-0.05,a_{1}=4$, and $a_{2}=\sqrt{2}$. Moreover, we assume that $v_{1}\left(t\right)=0$, $v_{2}\left(t\right)=0.01\sin\left(0.5t\right)$, $v_{3}\left(t\right)=0$, and $v_{4}\left(t\right)=0.01\cos\left(0.5t\right)$. The membership is selected as the same as in Example 1. And note that this is a continuous-time system. Thus, we need to discretize it. Through the Euler discretization method, (32) can be rewritten as

$$\begin{array}{c} x_{i}\left(\tau +1\right)=\left(I_{2}+\bar{T}A_{f_{i}}\right)x_{i}\left(t\right)+\bar{T}B_{f_{i}}\left(u_{i}\left(t\right)+v_{i}\left(t\right)\right), \end{array}$$

where $\bar{T}$ is sampling period, which is set to $\bar{T}=0.3$.

Then, by LMI (30), we can obtain $P_{1}$, $P_{2}$, $X_{1}$, and $X_{2}$:

$$\begin{array}{c} \begin{array}{c} P_{1}=\left[\begin{array}{cc} 76.7506 & 14.4825\\ 14.4825 & 17.6831 \end{array}\right],P_{2}=\left[\begin{array}{cc} 94.8937 & -19.6246\\ -19.6246 & 110.8308 \end{array}\right],\\ X_{1}=\left[\begin{array}{c} 66.1797\\ -75.3586 \end{array}\right],X_{2}=\left[\begin{array}{c} 76.5624\\ -87.2183 \end{array}\right]. \end{array} \end{array}$$

and we can calculate $M_{1}$ and $M_{2}$ as

$$\begin{array}{c} \begin{array}{c} M_{1}=\left[\begin{array}{c} 0.5780\\ -0.5776 \end{array}\right],M_{2}=\left[\begin{array}{c} 0.6686\\ -0.6686 \end{array}\right]. \end{array} \end{array}$$

**Figure 7:**
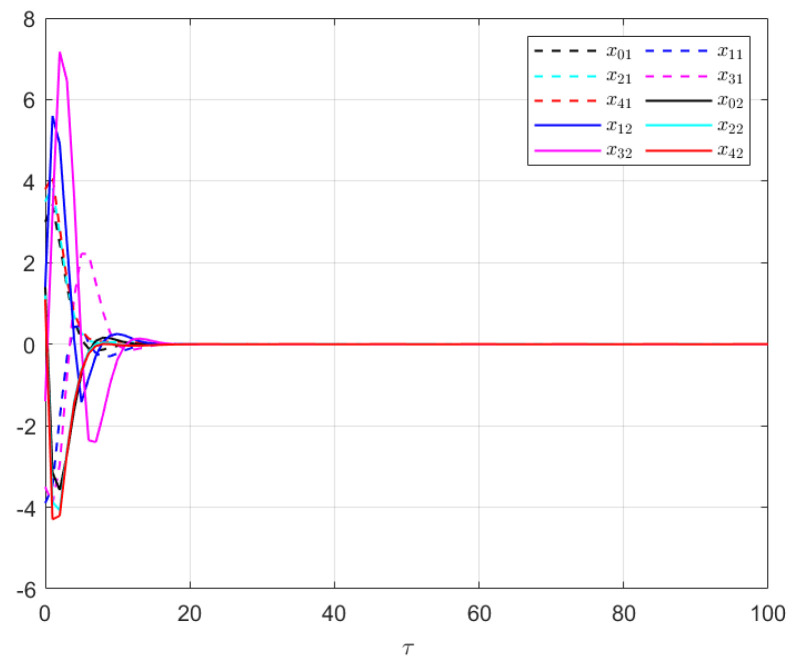
The UIO−based distributed control consensus performance in Example 2.

**Figure 8:**
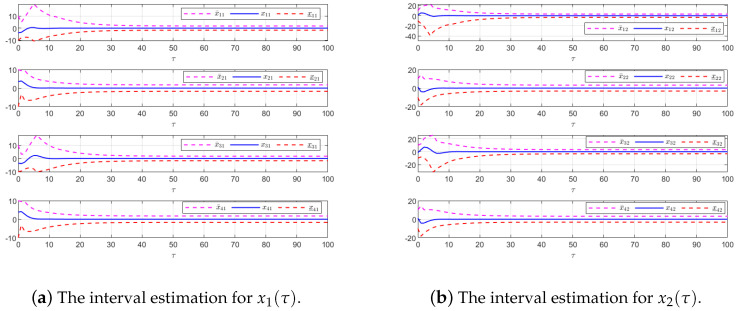
The interval estimation performance in Example 2.

**Figure 9:**
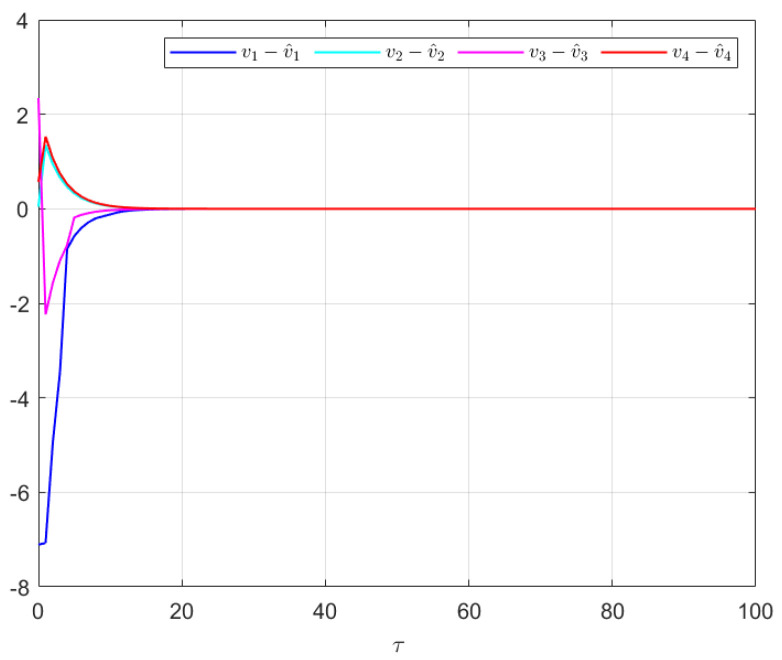
The UIR performance in Example 2.

**Figure 10:**
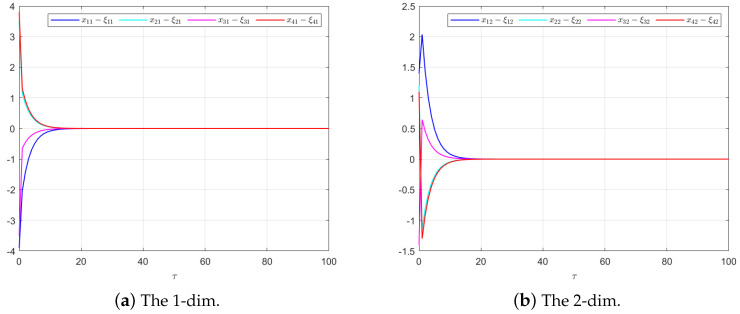
The state estimation performances for UIO (19) in Example 2.

**Figure 11:**
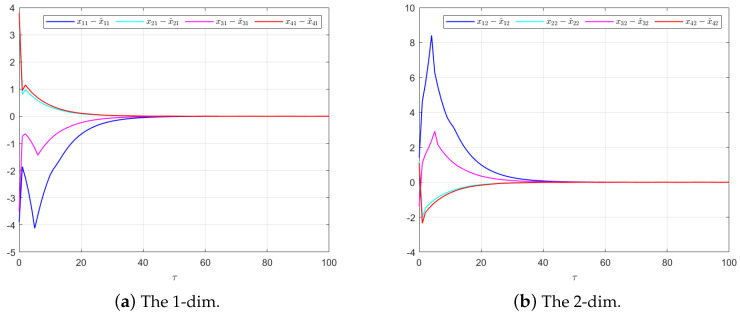
The state estimation performances for Luenberger-like Observer (4) in Example 2.

The effectiveness of the UIO-based distributed controller in (25) is demonstrated in Figure 7, Figure 8, Figure 9, Figure 10 and Figure 11. Figure 7 shows the state trajectories of the followers and leader, confirming that consensus is achieved asymptotically under the proposed control protocol, even in the presence of external disturbances in T–S fuzzy MASs. Figure 8 compares interval estimates with actual state trajectories, validating that the controller provides reliable state estimation within bounded intervals. Figure 9 illustrates the asymptotic convergence reconstruction of the UI using the proposed UIO framework. Figure 10 and Figure 11 highlight the asymptotic convergence of state estimates over time.

## 6. Conclusions

The work of the paper focuses on developing a UIO-based distributed control strategy for T–S fuzzy MASs with unknown inputs, aiming at accomplishing asymptotic convergence MAS consensus. In order to obtain both the asymptotic estimation of the system state and the asymptotic UIR, a UIO design methodology for a fuzzy T–S system is developed. The designed UIO contains two parts: a Luenberger-like state observer and a UIR, where the UIR part has the advantage of decoupling the control input. For constructing the UIO, an algebraic correlation of the UI and the state is given in advance through an interval estimation of the state, which is produced by the combination of a Luenberger-like observer and zonotope theory. Next, utilizing the state estimation and the UIR, a distributed control protocol is proposed, where the LMI technique plays a significant role in the design. And, under the proposed distributed control protocol, the consensus can be accomplished asymptotically for the T–S fuzzy MAS with UI. Further research should focus on introducing some event-triggered schemes into the designs.

## Data Availability

Data are contained within the article.
